# Determinants and efficiency of Pakistan’s chemical products’ exports: An application of stochastic frontier gravity model

**DOI:** 10.1371/journal.pone.0217210

**Published:** 2019-05-30

**Authors:** Rao Muhammad Atif, Haider Mahmood, Liu Haiyun, Haiou Mao

**Affiliations:** 1 Department of Economics, COMSATS University Islamabad, Lahore Campus, Lahore, Pakistan; 2 Department of Finance, College of Business Administration, Prince Sattam bin Abdulaziz University, Alkharj, Saudi Arabia; 3 School of Economics, Huazhong University of Science and Technology, Wuhan, China; 4 China Institute of Boundary and Ocean Studies in Wuhan University, School of Political Science and Public Administration Wuhan University, Wuhan, China; Universitat de Valencia, SPAIN

## Abstract

The estimation of efficiency of industry-specific exports is very important to find exports’ gap and to frame exports promotion policy for targeted industry. This study attempts to investigate the main determinants of chemical products’ exports of Pakistan with 62 trading partners by applying Stochastic Frontier Analysis (SFA) on an augmented gravity model for a period 1995–2015. The results corroborate that chemical products’ exports follow gravity patterns. This study finds a negative and significant impact of import tariff on exports of chemical products while the positive impact of devaluation has been observed. Further, the estimations also take into account the impact of Preferential Trade Agreements (PTA), colonial links, common language, political disputes and contiguity by incorporating dummy variable for each variable and the expected positive effects are found except an insignificant effect of Contiguity. Further, the negative impact of political disputes is observed. The exports’ efficiency analyses reveal that Pakistan’s chemical exports are well below the optimal level and there exists a huge untapped exports’ potential with its neighboring, Middle Eastern and European countries.

## Introduction

**Fig 1:**
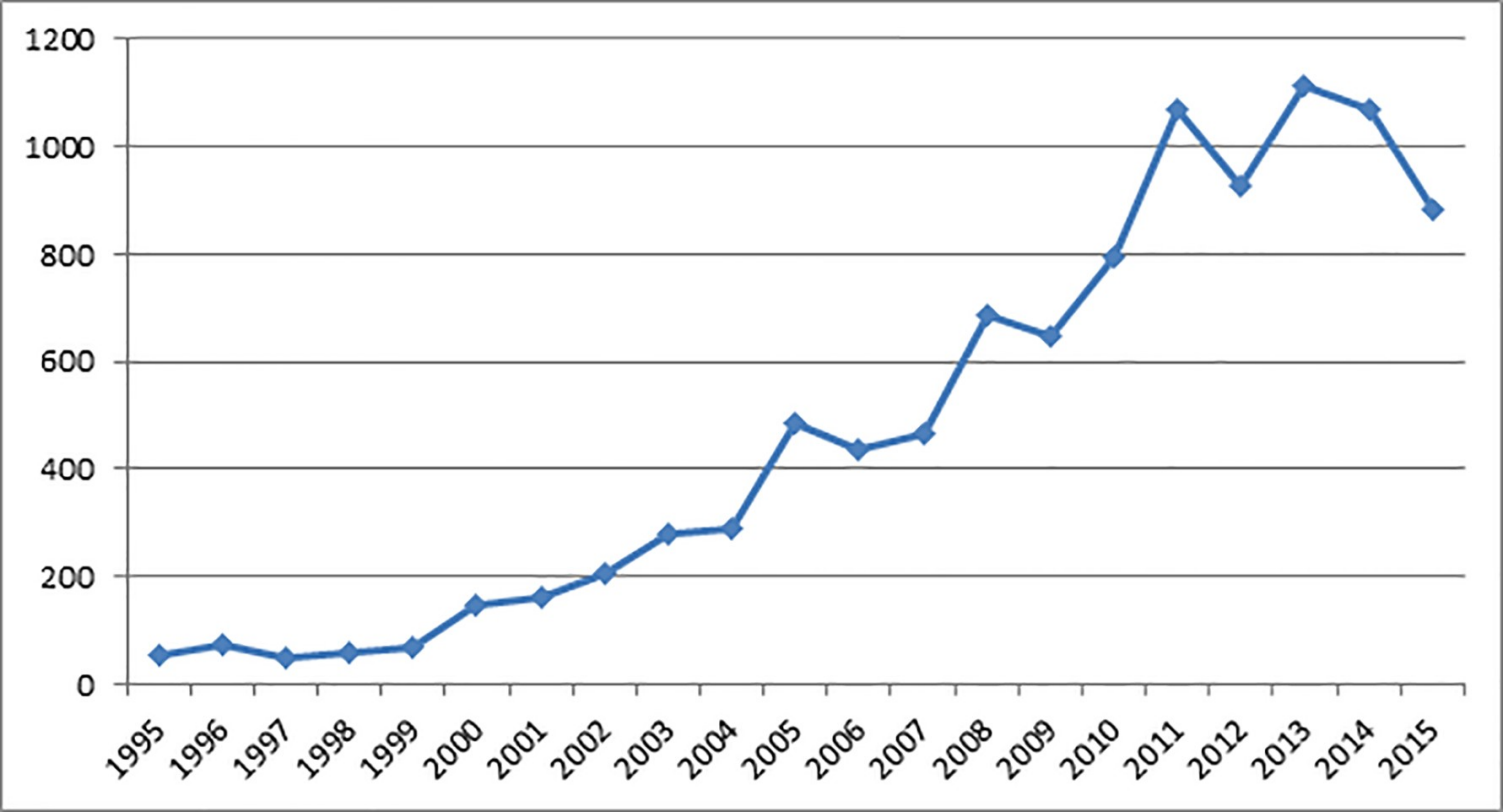
Trend of chemical products’ exports in Pakistan.

Pakistan has adopted trade liberalization policy in the early 1980s. The export-led growth policy has brought a significant rise in exports from 2,364 million dollar in 1980s to 23,667 million dollars in 2015. The composition of exports has also been changed significantly. The share of manufacturing exports was around 48% in 1980s and it has increased to 80% in 2015 [1]. The exports-support programs have not only increased the traditional exports but also brought new products in exports list of Pakistan in recent years. The chemical sector is an emerging sector in the international market. It is the third largest export sector of Pakistan after textile and agriculture sectors and second largest sector in the manufacturing exports. Fig 1 illustrates the trend of chemical products’ exports during 1995–2015.

On the average, Fig 1 describes a rising trend in chemical products’ exports of Pakistan during 1995–2015. However, some negative fluctuations may also be observed in chemical products’ exports. During 2011–15, chemical products’ exports have shown a declining trend with a single rise of year 2013. It should be a matter of concern for Pakistan whose economic growth largely depends on the external sector. Generally, export sector of any country depends on a combination of factors like supply side issues [2], bilateral trade costs [3] and external demand and market access strategies [4].

Table 1 shows that textile and clothing exports are contributing around 90% of total manufacturing exports of Pakistan during 2005–17. A good number of studies has been conducted to find the determinants of textiles’ exports [5,6]. After textile and clothing sector, chemical products’ sector is second largest contributor in the manufacturing exports of Pakistan. Further, the percentage trend is mostly increasing in recent years. Considering the importance of chemical sector, it is imperative to analyse the factors affecting the exports of chemical products. Additionally, the present study also attempts to exam the exports’ efficiency of chemical sector. Therefore, the purview of the present study includes (i) to determine the most significant factors of chemical products’ bilateral exports applying Stochastic Frontier Analysis (SFA) on an augmented gravity model. In addition, we examine the impact of border, cultural similarities and PTA on chemical products’ exports, (ii) to compute the efficiency and potential of chemical products’ exports of Pakistan with her trading partners. To best of our knowledge, the estimation of the efficiency of chemical products’ exports of Pakistan applying Stochastic Frontier Gravity Model (SFGM) is missing in the literature and we may claim for an empirical contribution in two ways. At first, we investigate the determinants of country-specific chemical products’ exports of Pakistan. Secondly, we apply a sophisticated technique SFGM to capture exports’ efficiencies. The application of SFGM is relatively scant in the trade literature.

**Table 1:** Percentage share in total manufacturing exports.

Year	Textile & Clothing	Chemical	Other Manufacturing Products
2005	92.68	4.21	3.11
2006	93.37	3.57	3.06
2007	89.35	3.73	6.92
2008	89.07	5.52	5.41
2009	89.62	5.89	4.49
2010	88.96	6.01	5.02
2011	89.12	6.98	3.90
2012	89.62	6.43	3.95
2013	89.49	7.18	3.34
2014	89.43	6.78	3.79
2015	90.93	6.05	3.02
2016	91.35	5.70	2.95
2017	90.03	6.97	3.00

Source: COMTRADE database

The rest of the study is structured as section-2 reviews the literature. Section-3 focuses on data, model and estimation strategy, section-4 illustrates the results and interpretations and section-5 concludes the findings of the study.

## Literature review

The determinants of exports have been analyzed in the trade literature considering either demand sided or supply sided or both. Investigating demand side factors, Senhadji and Montenegro [7] estimate exports’ demand function for 53 industrial and developing countries. Their estimates reveal that average income and price elasticities are found approximately 1.5 and -1 respectively. It indicates that exports are responding significantly to the income and price variables. But, they do not incorporate exchange rate factor in the relative price variable. Later on, several studies include exchange rate variable in the exports’ demand function. For instance, Achy and Sekkat [8] augment exchange rate in the exports’ demand function of Algeria, Egypt, Morocco, Tunisia and Turkey and find a significant contribution of exchange rate. Later on, some other studies also find a significant effect of exchange rate on exports [9,10,11,12].

Literature also considers supply sided factors like unit labor cost, productivity, subsidies and taxes to determine the exports at industry level and aggregate level [13,14,15]. The exports’ supply models have been criticized because of ignoring demand side factors. In fact, the isolated demand sided model or supply sided models are biased due to the omitted variable problem [16]. In line of this argument, Funke and Holly [17] claimed that exports’ model is not efficient if ignore either demand or supply sided factors. Later on, many studies care the both demand and supply sided variables in the exports’ models [4,16,18,19].

Using gravity model, Mao et al. [20] found the positive effects of belt and road initiative, facilities reducing trade barriers on the node countries to China. In the sector specific exports’ literature, some studies have investigated the determinants of chemical products’ exports. For example, Roy [21] investigate the demand and supply sided factors of manufacturing sectors’ exports in India. He reports that exchange rate is found as most significant factor of chemical products’ exports. Using a period 1996–2001, Athanasoglou et al. [22] find a high magnitude of price competiveness elasticity of chemical sector’s exports of Greek. It indicates that Greek chemical products’ exports are mainly driven by relatively lower price in the foreign market. Guloglu and Bayar [16] incorporate both demand and supply factors in gravity equation to find out the determinants of chemical products’ exports of Turkey along with other industries’ analysis. Their findings reveal that trading partner’s income, exchange rate and domestic productivity are found as important determinants of chemical products’ exports.

Some empirical studies have also been reviewed which have explained the exports’ behavior of Pakistan. For example, Hassan et al. [23] find that depreciation and income per capita have improved the trade deficit of Pakistan. However, money supply has been found responsible for increasing trade deficit. On the other hand, money demand and supply may also be affected by exchange rate [24] and this may also have indirect effect on exports. Gul and Yasin [25] apply gravity trade model incorporating border, trade and cultural links in the analysis. Their findings suggest that aggregate exports with major trading partners of Pakistan follow gravity patterns. For example, they find that trade is inversely related with distance between trading partners and positively associated with trading partners’ Gross Domestic Product (GDP). Further, the positive impacts of border, bilateral trade relations and cultural similarities have also been reported.

SFGM has been utilized in the trade literature to find the exports’ efficiency. At first, Kalirajan [26] investigates the exports’ performance of Australia with her trading partners using SFGM. He finds that Australian exports have been increased by 15% after trade agreements. Ravishankar and Stack [27] compare the actual and potential trade of Eastern and Western European countries to investigate the level of integration between East-West trade. They find that their actual trade is approximately 67% of the potential trade and they claim that trade expands because of the low level of resistance in East-West trade. Nasir and Kalirajan [28] investigate the service sector exports’ potential for emerging and relatively developed Asian countries in the information technology, professional and telecommunication sectors using SFGM and compare the trade potentials with the North American and European countries. Their findings suggest that Asian countries are far-away from the exports’ potential with developed countries. Therefore, they suggest to adopt latest technologies in the service sector’s exports to improve the exports’ potential with developed world. In case of Pakistan, Atif et al. [29] apply SFGM to analyze the determinants of agriculture exports of Pakistan with her 63 trading partners. Apart from traditional gravity variables, they also incorporate bilateral exchange rates, tariff rates, PTA, common border and colonial links. Their findings reveal that tariff and distance have negative effects whereas GDP of exporter and importer, bilateral exchange rates, PTA and common border have positive effects on the agriculture exports of Pakistan. In the analysis of agriculture exports’ efficiency, they find that actual exports are lesser than potential exports with all trading partners. Further, they report that the highest agriculture exports’ gap of Pakistan is found with her neighboring partner India.

The literature reflects the importance of gravity model in estimating the bilateral trade at commodity level. Further, some studies also confirm the importance of SFGM to estimate the trade efficiencies. However, the use of SFGM is still relatively scant in the trade literature. Therefore, this present study contributes in Pakistan’s trade literature by investigating SFGM for chemical products’ exports considering both demand and supply sided factors and is going to attract the attention of researchers to use SFGM at commodity level trade.

## Model, methodology and data

Gravity model is one of the famous models to determine the international trade flows and to explain the movements of factors of production. Tinbergen [30] proposed and subsequently Anderson [31] extended this model on the assumptions that bilateral trade is directly proportional to the products of partners’ GDP and has an inverse proportional relationship with the bilateral distance. The basic gravity model in log-linear form can be expressed for chemical products’ exports in our case as follows:

$$\ln Xc_{hjt}=\alpha +\beta \ln Yr_{ht}+ο\ln Yp_{jt}+\delta \ln Dist_{hj}+\varepsilon_{hjt}$$ (1)

Where, “Xc” is the value of chemical products’ exports from Pakistan to its trading partners. “Yr” is the GDP of Pakistan and “Yp” is the GDP of Pakistan’s trading partner. “Dist” stands for distance between Pakistan and trading partner. “*ε*” is the error term. “h” denotes home country Pakistan. “j” = 1….62 is for trading partners and t = 1995….2015 annual series. “ln” stand for natural log.

Gravity model has evolved tremendously in order to provide more clear and robust estimates. For instance, Rauch [32] asserts that cultural and language similarities and contiguity attributes should be incorporated in the gravity model to explain trade patterns. Further, Frankel et al. [33] suggest to incorporate trade agreements in the gravity model and Cho et al. [34] augment the gravity model with exchange rate.

We extend the traditional gravity model incorporating demand and supply side factors and also incorporate exports’ stimulating and resisting variables. The augmented gravity model for chemical products’ exports is as follows:

lnXchjt=α+βlnYrht+οlnYpjt+δlnDisthj+ϕ1ln(1+Imtjt)+ϕ2lnBxrhjt+ϕ3Conhj+ϕ4Colhj+ϕ5Ptahjt+ϕ6Pdhj+ϕ7Langhj+ζhjt (2)

Where, “Imt” is the importing country’s tariff rate on Pakistan’s chemical products’ exports. “Bxr” is the bilateral exchange rate between Pakistan and importing country. “Con”, “Col”, “Pta”, “Pd” and “Lang” stand for contiguity, colonial links, preferential trade agreements, political disputes and common language respectively.

Since its inception, the gravity model has become a workhorse to explain international trade flows. Leamer and Levinsohn [35] claim that gravity model is suitable in providing robust findings in empirical testing of trade models. But, Anderson and van Wincoop [36] criticize the model that it does not explain the effect of Multilateral Trade Resistance (MTR) on the bilateral trade. The MTR includes the factors like tariff rate trade agreements, distance, contiguity, common language and colonial links [37]. Ravishankar and Stack [27] state that the typical gravity estimates are drawn from the data set of those countries that have normal trade-relationships. The major disadvantage of this methodology is that the prospects are defined based on sample averages instead of the maximum possible frontiers.

At first, Kalirajan [26] proposes SFGM by applying the SFA on the gravity model. The SFA technique was developed by Aigner et al. [38] in the production economics. The SFA posits a Production Possibility Frontier (PPF) and it estimates a maximum level of output which can be achieved from a given level of inputs. A firm/industry operating on the PPF is considered as technically efficient whereas inefficient firm operates blow the frontier output. Therefore, the latter refers the possibility of further expansion of output. Kalirajan [26] suggests that SFGM may be utilized; even a model has not sufficient information about the omitted variables. Secondly, it calculates the impact of economic distance term which may cause non-normality and heteroskedasticity and it also isolates the analysis from statistical white noise term. Thirdly, it provides exports’ efficiency and potential.

The insertion of SFA in the gravity exports’ equation allows the model to quantify the potential exports at bilateral level. These exports’ frontier quantities are influenced by a random error which may either be negative or positive and thus allows stochastic frontier exports to fluctuate around the deterministic part of the model [27]. Afterward, a comparison can be made between actual exports and the forecasted optimal exports to calculate the gap. The strong theoretical and policy relevance of SFA results provide a good justification to use it. Therefore, the Eq (2) of typical gravity model incorporating SFA may be transformed into SFGM as follows:

lnXchjt=α+βlnYrht+οlnYpjt+δlnDisthj+ϕ1ln(1+Imtjt)+ϕ2lnBxrhjt+ϕ3Conhj+ϕ4Colhj+ϕ5Ptahjt+ϕ6Pdhj+ϕ7Langhj+ehjt−vhjt (3)

The Eq (3) is same as Eq (2) except the error term “*ζ_hjt_*” which is divided into “e_hjt_” and “v_hjt_”. The e_hjt_ is a double-sided error-term and is showing a statistical noise because of estimation errors with an assumption of N(0~σ^2^_e_). The v_hjt_ is one-sided error-term and is truncated at zero. It is independent of e_hjt_ and regressors. It is a positive random variable which measures the exports’ inefficiency and its value may vary between 0 and 1. The zero value implies that actual exports are equal to potential exports. Hence, there is no statistical error and the impact of omitting variable is negligible. When, it takes the value other than 0 (i.e. v_hjt_ is less than or equal to 1). It implies that the impacts of omitting variables are significant which could impede exports. Thus, v_hjt_ represents deviation from optimal exports’ level. It may take place due to MTR which are quite tough to quantify and thus result in inefficient exports’ performance.

The Eq (3) can be considered as pooled frontier [38] and we may apply maximum likelyhood methodology to estimate the parameters’ values. This technique also generates the diagnostic parameters whose values would justify the use of SFA. Moreover, the null hypothesis (σ^2^_v_ = 0) can be tested against the alternate hypothesis (σ^2^_v_>0) to estimate technical efficiencies. The rejection of this hypothesis may confirm the appropriateness of SFA. For the estimation of technical efficiency, we are following the Battese and Coelli [39]:

$$E\left[\exp(-v_{hjt})|e_{hjt}+v_{hjt}\right]=\left[\frac{1-\Phi \left[\sigma_{\alpha }+\gamma (e_{hjt}+v_{ijt})/\sigma_{\alpha }\right]}{1-\Phi \gamma (e_{hjt}+v_{hjt})/\sigma_{\alpha }}\right]\times \exp\left[\gamma (e_{hjt}+v_{hjt})+\frac{\sigma_{\alpha }^{2}}{2}\right]$$ (4)

The density function has been defined by Φ(.). The estimated efficiency (γ) from the Eq (4) can be varied from 0 to 1. Here, the efficiency score = 0 is signaling for inefficiency and further trade is possible with the given determinates of trade in Eq (3). Further, efficiency score = 1 is a proof for a perfect efficiency and actual trade is matching exactly the potential possible trade in this case.

There are other alternative measures to verify estimates of gravity model. The Fixed Effect (FE) method is another frequently used technique to obtain gravity estimates [40,41]. This approach accounts for unobservable heterogeneity and controls MTR phenomenon in the gravity model [42]. However, the FE technique does not estimate the time invariant variables of the gravity equation as such variables are eliminated by the averaging with transformation. The variables like distance and contiguity are usually rule out during the estimation. This study also uses FE technique to confirm the gravity model coefficients of our proposed model. FE equation considering unobserved cross sectional heterogeneity is as follows:

lnXchjt=ι0hj+ι1lnYrht+ι2lnYpjt+ι3ln(1+Imtjt)+ι4lnBxrhjt+ι5Ptahjt+ψhjt (5)

### Rational for variables

GDP has been used as a proxy for market size of a country. Exporter’s and importer’s market size are expected to have positive effects on the exports. The Pakistan’s market size is reflecting the exports’ capacity of chemical products and importer’s market size is showing demand for chemical products’ exports of Pakistan. If estimated coefficient of GDP of exporting country is estimated greater than the coefficient of the GDP of importing country, then the exporting country may have home effect and turn out to be a net exporter [43]. Therefore, this study also attempts to find out home country effect for chemical products’ exports.

Exports are significantly affected by the transportation cost. The transportation cost includes shipping cost, interaction cost and time related cost. Since the calculation of these costs is complex. So, geographical distance is a renowned proxy for it. Further, tariff rate of importing partners obstructs export flows and it is another addition to exports’ cost. Atif et al. [29] find that higher tariff rates hinder exports’ flows. Therefore, we also regress the bilateral tariff rates on chemical products’ exports of Pakistan. In gravity model, Sertić et al. [4] and Atif et al. [29] claim that insertion of exchange rate factor remains useful to explain the trade flows at bilateral level. This study uses bilateral exchange rate defined as ratio of Pak. Rupees per US dollar to importing country’s currency per US dollar. Thus it means that the increasing ratio is showing a depreciation of Pak. Rupee which may have positive effect on the exports.

Countries sharing common borders are expected to do more trade [29] and this may be captured by a dummy variable assuming 1 for border country and 0 otherwise. Further, trading partners usually do the PTA to promote regional trade to prefer the member countries of agreement over non-member countries. This variable is also captured by a dummy variable assuming 1 for PTA member country and 0 otherwise. Different Languages may be claimed for a trade barrier and common language may enhance the bilateral trade due to communication’s easiness. Therefore, a dummy variable is utilized by assuming 1 for common language country and 0 otherwise. The positive contributions of PTA, common language and common borders are expected on the chemical exports.

In addition, we use Baier and Bergrstrand [37] methodology to examine MTR Phenomenon. It is a better technique because it includes both time variant and invariant trade resisting factors without taking dummies. In our analysis, all the trade resisting factors like distance, tariff rates, trade agreements, contiguity, cultural similarities and colonial links have been adjusted for MTR by using Baier and Bergrstrand [37] methodology.

### Data source

This study utilizes a panel data of bilateral chemical products’ exports of Pakistan with her 62 trading partners for a period 1995–2015. The maximum possible frequency of data has been used, both in terms of time and trading partners. All the variables, except dummy variables, are taken in the natural log form to analyze the relationships. The data sources and description has been presented in Table 2.

**Table 2:** Variables’ descriptions and sources.

S#	Variable	Data Description	Data Source
1	Chemical exports at market price	The sum of chemical products 51 to 59 from SITC-Rev.3 classification.	COMTRADE database Author’s Calculation
2	Exporter & importers GDP	Gross Domestic Product at market price	WDI Database
3	Bilateral exchange rate	The ratio of Pak. Rupees per US dollar to importing country’s currency per US dollar (based on Purchasing power Parity)	WDI Database
4	Import tariff	1+(import tariff/100)	World Integrated Trade Solution database (WITS)
5	Distance	Crow flies distance between trading partner’s capital	CEPII database
6	Border	A dummy variable to capture common border effect	CEPII database
7	Preferential Trade Agreements	A binary series which take the value of ‘1’ if the export destination has granted any preferential market access to Pakistan and zero otherwise.	CEPII database
8	Colonial Links	A binary variable which assumes the value of ‘1’ if both trading partners share same colonizer UK before 1945 and 0 otherwise	CEPII database
9	Common Language	A binary series which possesses the value of ‘1’ if the trading partners have similar official language and zero otherwise.	CEPII database
10	Political Disputes	A dummy variable which takes the value of 1 if trading partner involved in war and zero otherwise.	CEPII database

## Empirical analyses

The descriptive statistics of all variables of our model show in Table 3. There are some important observations in this table. First, the data is unbalanced. Second, virtually none of these series deviates substantially from normal distribution. Third, the importer GDP, bilateral exchange rate and tariff rate have a well range of minimum and maximum observations. It indicates that trading partners are quite heterogeneous. Fourth, the correlation matrix confirms that there is no multicollinearity in the model.

**Table 3:** Descriptive statistics.

S#	Variable	Obs.	Mean	S.D.	Min	Max	1	2	3	4	5	6	7	8	9	10	11
1	lnXc	1239	14.32	2.12	4.97	18.8	1										
2	lnYr	1282	25.5	0.53	24.82	26.3	0.19	1									
3	lnYp	1267	26.1	1.92	20.38	31.1	0.23	0.02	1								
4	lndist	1282	8.39	0.58	6.69	9.56	-0.3	-0.06	0.32	1							
5	lnBxr	1237	0.8	0.59	-0.64	2.22	0.04	-0.08	-0.2	0.04	1						
6	ln(1+Imt)	968	0.06	0.07	0	0.32	-0.1	-0.17	-0.4	-0.18	-0.24	1					
7	Coli	1282	0.33	0.47	0	0.99	0.14	0.01	-0.3	-0.3	-0.09	0.1	1				
8	Pta	1282	0.15	0.36	0	0.99	0.17	-0.03	-0.1	-0.37	-0.21	0.3	0.3	1			
9	Con	1282	0.06	0.23	0	0.99	0.19	-0.01	0.16	-0.35	-0.08	0.2	0	0.53	1		
10	Pd	1282	0.02	0.16	0	0.99	0.12	0.01	0.04	-0.43	-0.06	0.2	0.1	0.36	0.7	1	
11	Lang	1282	0.26	0.43	0	0.99	0.13	-0.02	-0	0.13	-0.09	0.1	0.3	-0.1	0	0.1	1

Source: Author’s calculation.

Table 4 shows the determinants of chemical products’ exports of Pakistan by using SFGM. The suitability of SFGM model has corroborated by multiple tests. First, rejection of null hypothesis (σ^2^_v_ = 0) against the alternative (σ^2^_v_>0) at 1% significance level and secondly the higher value of LR test is supporting this technique. Thirdly, the statically significant coefficient of gamma (γ) also shows that the use of SFGM technique is suitable for our model which is close to 1 (0.75). The “γ” measures the ratio of variations in exports caused by exports’ inefficiency to total variations in exports. It refers the presence of some country-specific constraints which are not measured by our proposed model. The “*η*” carries negative sign which explains that exports’ inefficiencies are increasing over time. The variance *σ*^2^ is statistically significant and other diagnostics are also fine to proceed.

**Table 4:** Gravity model regression results.

	Maximum Likelihood coefficients from SFGM		Fixed Effect Model	
Variable	Coefficient.	Std. Err.	Coefficient.	Std.Err
lnYr	1.74***	0.24	1.48**	0.59
lnYp	0.43***	0.07	0.46***	0.14
lndist	-1.12***	0.27		
lnBxr	0.07*	0.04	0.31*	0.16
ln(1+Imt)	-2.93***	1.02	-2.02**	0.94
Col	1.20***	0.26		
Pta	0.77**	0.37	0.93***	0.18
Con	0.41	0.73		
Pd	-2.80***	0.93		
Lang	0.62**	0.25		
Intercept	-28.91***	6.2	-36.55**	15.7
(γ)	0.75	0.04		
μ	6.04	1.04		
(*η*)	-0.07	0.01		
*σ*^2^	6.25	1.1		
*σ*^2^_v_	4.68	1.02		
*σ*^2^_e_	1.57	0.08		
LR test	-1626.16			
χ^2^	170.51			
R^2^			0.43	

Source: Author’s Calculations

*, ** and *** represent the level of significance at 10%, 5% and 1% respectively.

The coefficients of SFGM exhibit the expected signs and are proved to be consistent with the theoretical prediction. The both coefficients of GDP of importer and of exporter are positive and highly significant. Therefore, income of both countries are supporting the sufficient demand and supply conditions of the chemical products. However, the magnitude of GDP of Pakistan is quite higher as compared to importing counterpart. Therefore, the response of supply with any change in GDP of Pakistan is larger than the response of demand due to change in GDP of importing countries. This finding has interesting implication that Pakistan is showing the greater power of adjustment of the supply of chemical exports in response of any change in demand from importing countries to maintain equilibrium condition of chemical exports. Moreover, the coefficients indicate that 1% increase in GDP of Pakistan increases exports by 1.74% whereas 1% increase in importing country GDP enhances the exports by 0.43%. Since, the estimated coefficient of Pakistan’s GDP is greater than the coefficient of importing countries’ GDP. Therefore we can claim that chemical products’ exports of Pakistan carry home country effect. The coefficient of distance with negative sign shows that 1% decrease in distance may increase the exports by 1.12%. Thus, the results corroborate that chemical products’ exports of Pakistan follow gravity patterns. This result also indicates that Pakistan should search nearby market to expand her chemical exports.

Apart from the basic gravity estimates, the other variables also give useful information. For instance, the statistically significant negative coefficient of tariff rate indicates that 1% increment of tariff rate may reduce the chemical exports by 2.93%. This indicates that exports related cost has quite significant impact on the exports. Therefore, Pakistan should develop the better trading relationship with the importing countries to reduce the tariff rates on the Pakistani chemical exports to accelerate the chemical exports. The coefficient of lnBxr shows that its 1% increment (depreciation of Pak. Rupee) may enhance the chemical products’ exports by 0.07%. So, the Pakistan needs to maintain or to appreciate the exchange rate because depreciation is expected to decline the exports revenue because of very low elasticity. Further, this estimated outcome is following the findings of Athanasoglou et al. [21] and Guloglu and Bayar [16].

This study has also added five dummy variables to find out the impact of contiguity, bilateral disputes, colonial links, PTA and common language on the chemical products’ exports. The coefficient of “Pta” is statistically significant which indicates that regional trade integration policies have positive impact on chemical products’ exports of Pakistan. Gul and Yasin [24] and Atif et al. [29] have also observed the same impact of PTA on total exports and agriculture exports of Pakistan respectively. This result also corroborates our finding of the effects of tariff rates as PTA are expected to reduce the tariff rates and lowering tariff rates may have positive contribution in the exports. The coefficients of colonial links and common language dummies have also proved to be statistically significant and confirm that historical relationships and cultural similarities have positive effects on chemical products’ exports of Pakistan. The negative and significant impact of political disputes (Pd) refers that political conflicts are found harmful for chemical products’ exports. The results of Lang and Pd recalls that Pakistan may boost its chemical exports if resolve the political problem with India which also has common language.

Finally, the contiguity is showing statistically insignificant effect. This refers that chemical products’ exports are not affected by border effect. Although it is quite unusual as compare to existing literature but there are many reasons for insignificancy of this effect. Pakistan has common border with Afghanistan, China, Iran and India. The most of Pakistan’s border is shared with India and both countries have historical political disputes. Secondly, Afghanistan is not economically and political stable. Thirdly, Iran is facing economic sanction by United Nations which impedes Pakistan chemical products’ exports to Iran. These facts can be claimed as potential reasons of Pakistan’s exports’ limitations to its neighboring partners. Table 4 also reports the estimates from FE methods. The signs of the explanatory series are quite similar to the SFGM estimates which confirm the validity of our model. However, some variations are observed in terms of magnitude and significance of variables. For instance, the coefficient of Pakistan’s GDP is found 1.48 which is smaller than the coefficient from SFGM. Whereas the coefficient of bilateral exchange is found quite higher in the FE estimates than the corresponding SFGM estimates. But, we prefer the estimates of SFGM for generalization due to its superiority over FE estimates.

The average technical efficiency drawn from SFGM has been demonstrated in Table 5. It represents the average exports’ performance with major trading partners. The estimates refer that Pakistan’s chemical products’ exports performance has not reached to optimal level (100% efficient) to any of the trading partner. It illustrates that there exists a great scope of improving chemical products’ exports’ performance with trading counterparts. For instance, exports’ efficiency of Pakistan is very low with its neighboring countries like India, Iran and China which are 5.1%, 2.57% and 11.37% respectively. So, improved trading relationships, decreasing political disputes and doing trade agreements with the neighboring countries may help in increasing the chemical exports. Moreover, a bleak Pakistan’s exports’ performance has been observed with big economies like Australia, Japan, Russia, Sweden, Indonesia and United Kingdom which are detected as 2.42%, 3.06%, 2.06%, 1.49%, 3.30% and 5.85% respectively. Therefore, increasing trading relationships with the developed world may accelerate the chemical exports. On contrary, highest exports’ efficiency has been calculated for Afghanistan which is 74.06% followed by 30.96% and 25.34% for Netherland and Turkey & Philippines respectively.

**Table 5:** Technical efficiency of chemical products’ exports with trading partners.

S.#	Partner	Technical Efficiency (%)	S.#	Partner	Technical Efficiency (%)
1	Afghanistan	74.06	32	Bahrain	5.05
2	Netherlands	30.96	33	Poland	4.88
3	Turkey	25.34	34	Mozambique	4.75
4	Philippines	25.34	35	Malaysia	4.71
5	Italy	17.73	36	Tanzania	4.32
6	Djibouti	16.78	37	Switzerland	3.81
7	Nigeria	15.54	38	Algeria	3.46
8	Rep. of Korea	14.21	39	UAE	3.42
9	USA	14.06	40	Indonesia	3.30
10	France	11.83	41	Maldives	3.24
11	Saudi Arabia	11.82	42	Jordan	3.22
12	China	11.37	43	Japan	3.06
13	Belgium	11.12	44	Ukraine	2.62
14	Germany	10.85	45	Iran	2.57
15	Egypt	10.49	46	Mauritius	2.51
16	Thailand	10.10	47	Argentina	2.48
17	Sri Lanka	9.46	48	Australia	2.42
18	Viet Nam	8.61	49	Russian Federation	2.06
19	Canada	8.20	50	Yemen	1.99
20	South Africa	8.15	51	Uganda	1.77
21	Singapore	7.92	52	Denmark	1.71
22	Bangladesh	7.40	53	Mexico	1.51
23	Greece	7.00	54	Sweden	1.49
24	Lebanon	6.58	55	Norway	1.48
25	United Kingdom	5.85	56	New Zealand	1.01
26	Spain	5.71	57	Kuwait	0.60
27	Kenya	5.71	58	Kazakhstan	0.58
28	Madagascar	5.64	59	Qatar	0.48
29	Oman	5.62	60	Mauritania	0.46
30	India	5.31	61	Iraq	0.00
31	Tunisia	5.17	62	Uzbekistan	0.00

Source: Author’s Calculations

Table 6 presents average exports’ potential and average exports’ gap estimated from SFGM. The exports’ gap has been estimated by a difference between potential exports and actual exports. The estimated gaps indicate that there exists a large exports’ potential for Pakistan’s chemical products with trading partners. For instance, Indian market has highest exports’ potential (2709.88 million dollar) for Pakistan’s chemical products. Such potential may exist due to, big market size of India and cultural similarities between partners. Pakistan has the second largest exports’ potential with UAE followed by Qatar and Kuwait which are 2466.89, 2348.29 and 1494.84 million dollars respectively. Table 6 portrays the negative signs of exports’ gaps with all the trading partners. This indicates that Pakistan’s chemical products’ exports with its trading partners are less than the optimal level in case of all trading partners. Therefore, the better trading policies may help Pakistan in boosting the chemical exports and it may contribute significantly in overall exports revenues as well.

**Table 6:** Pakistan’s chemical products exports’ gap.

S.#	Trading Partner	Actual exports	Potential Exports	Exports gap	S.#	Trading Partner	Actual exports	Potential Exports	Exports gap
1	India	17.72	2727.60	-2709.88	32	Egypt	6.98	280.94	-273.96
2	UAE	43.82	2510.71	-2466.89	33	Tanzania	1.54	267.28	-265.73
3	Qatar	0.61	2348.90	-2348.29	34	Saudi Arabia	7.77	245.47	-237.71
4	Kuwait	1.18	1496.02	-1494.84	35	Argentina	0.20	241.32	-241.12
5	Spain	7.42	1281.65	-1274.23	36	Greece	2.00	237.78	-235.77
6	China	30.33	1192.88	-1162.54	37	Lebanon	1.29	235.81	-234.51
7	Bangladesh	12.13	1071.08	-1058.95	38	Uganda	1.02	208.11	-207.10
8	Rep. of Korea	32.82	1069.26	-1036.44	39	Switzerland	0.77	204.07	-203.30
9	Malaysia	4.16	995.65	-991.48	40	Sweden	0.14	197.64	-197.50
10	Jordan	1.82	847.96	-846.14	41	Germany	5.34	184.62	-179.28
11	United Kingdom	7.80	781.44	-773.64	42	Norway	0.18	169.82	-169.64
12	Kazakhstan	1.25	741.95	-740.70	43	Denmark	0.12	154.26	-154.14
13	Indonesia	1.20	653.81	-652.60	44	Mexico	0.08	145.25	-145.17
14	Oman	4.07	609.43	-605.36	45	Thailand	5.22	138.85	-133.64
15	Iran	2.83	599.98	-597.16	46	France	6.63	132.47	-125.84
16	Ukraine	3.89	599.33	-595.44	47	Nigeria	9.24	131.91	-122.67
17	Japan	2.66	570.48	-567.82	48	Belgium	9.01	130.96	-121.96
18	Mauritius	0.39	570.05	-569.65	49	South Africa	4.14	122.82	-118.68
19	Russian Federation	0.79	538.24	-537.45	50	Netherlands	20.42	116.70	-96.28
20	Singapore	6.05	470.93	-464.88	51	Afghanistan	79.91	109.70	-29.79
21	Sri Lanka	14.90	469.61	-454.71	52	Philippines	15.49	107.54	-92.05
22	Poland	4.08	446.74	-442.66	53	New Zealand	0.04	95.40	-95.36
23	Canada	2.97	427.73	-424.76	54	Algeria	0.32	91.44	-91.12
24	USA	13.98	384.90	-370.92	55	Maldives	0.67	46.28	-45.61
25	Kenya	4.48	380.95	-376.47	56	Madagascar	0.32	43.02	-42.70
26	Yemen	1.76	370.93	-369.17	57	Tunisia	0.65	33.59	-32.93
27	Bahrain	1.67	351.34	-349.67	58	Mauritania	0.15	23.26	-23.11
28	Italy	29.97	323.00	-293.02	59	Mozambique	0.54	22.05	-21.51
29	Turkey	41.53	315.77	-274.23	60	Djibouti	0.62	7.17	-6.55
30	Australia	0.69	310.64	-309.94	61	Iraq*	1.49		
31	Viet Nam	7.00	291.53	-284.53	62	Uzbekistan*	0.98		

Source: Author’s Calculation. All figures are in average US million dollars.

*indicates that exports potential and exports gap could not be estimated.

## Conclusions

This study uses SFA to estimate gravity equation for Pakistan’s chemical products’ exports for a panel of 62 countries and a time period of 1995–2015. This study augments the traditional gravity model by incorporating some demand and supply sided determinants of chemical products’ exports of Pakistan. The estimates from SFGM reveal that Pakistan’s chemical products’ exports follow gravity pattern. The larger coefficient of Pakistan’s GDP as compared to importers’ GDP confirms the existence of home effect. This study finds that chemical products’ exports are highly sensitive to tariff rates. This study also finds that devaluation of Pak. Rupee may help in boosting the chemical products’ exports. The positive and statistically significant impacts of PTA, colonial links and cultural similarities have been observed. On the other hand, political disputes are reducing chemical products’ exports significantly. Further, this study quantifies the exports’ potential by estimating technical efficiency with the help of SFA. The technical efficiency estimates indicate that exports’ performance is less than 30% with 60 trading partners. Therefore, the actual chemical products’ exports are significantly lower than the potential exports.

Considering this research’s findings, the study proposes following suggestions. This study finds a positive but insignificant impact of common border which indicates that Pakistan is not enjoying the benefit of trade from neighboring countries. Thus, the political and trade authorities should engage the neighboring counterparts to resolve political and trading disputes to increase the maximum possible chemical products’ exports as political disputes are also responsible for reducing these in empirical findings. Secondly, the effects of PTA and colonial links exhibit high elasticities which refer that these factors must be taken under consideration while doing trading agreements to increase exports of Chemical products of Pakistan. Thirdly, the negative effect of distance and positive effect of language suggest that Pakistan should focus on the nearby countries’ markets with common language to increase the chemicals’ products exports. Moreover, the positive bordering effect recalls that Pakistan has very large size of countries like China and India. The increasing trading linkages with these countries may boost the chemical exports at a high pace. Fourthly, the exchange rate stability or appreciation may help in increasing the exports’ revenues as a very low exchange rate elasticity is estimated and devaluation policy is not fruitful in this case for exports’ revenues.

## Supporting information

S1 File Data series. (XLSX)

S2 File Supplementary material. (DOCX)
