# Supplementary material for: Determinants and efficiency of Pakistan’s chemical products’ exports: An application of stochastic frontier gravity model
Source: PLoS One. 2019 May 30;14(5):e0217210. doi: 10.1371/journal.pone.0217210 (PMC6542523; doi:10.1371/journal.pone.0217210)
Supplement: S2 File — (DOCX) [file pone.0217210.s002.docx]

Appendix-A

The linear gravity equation estimated by Anderson and van Wincoop (2003) is as follows:

$$X_{hj}=\ln y_{r}+\mathrm{lny}_{p}+\ln y_{w}+(1-\sigma)\left[\mathrm{t}_{hj}+\ln P_{h}+\ln P_{j}\right]+e_{hj}.....(1)$$

Where, X_hj_ = the nominal value of exports from country “h” to “j”, y_h_ & y_j_ = the nominal income of respective country, Y_w_ = world Income, t_ij_ = trade costs between country “h” & “j”, Ph &Pj = multilateral resistance (MR) terms for country “h” & “j” respectively. Finally, σ is the elasticity of substitution between all goods.

Where

$$P_{h}=\sum_{h=1}^{c} \left[\frac{t_{hj}}{p_{j}}\right]^{1-\sigma}\frac{y_{j}}{y_{k}}.................(2)$$

$$P_{j}=\sum_{j=1}^{c} \left[\frac{t_{hj}}{p_{h}}\right]^{1-\sigma}\frac{y_{h}}{y_{k}}.................(3)$$

Baier and Bergstrand (2009) develop an approach that takes into account for arbitrary distribution of both inward and outward MR without the insertion of fixed effect. Their approach follows first order Taylor series approximation of the two non-linear MR terms. This technique make enables us to consistently estimate gravity model what Concretely, Baier and Bergstrand (2009) show that the following model produces similar estimates from those obtained by fixed effect method but without taking into account of dummy variables:

$$X_{hj}=\ln y_{h}+\mathrm{lny}_{j}+\ln y_{w}+(1-\sigma)\left[\mathrm{t}_{hj}*\right]..............(4)$$

Where

$$t_{hj}*=\mathrm{lnt}_{hj}-\sum_{j=1}^{N} \theta_{j}\mathrm{lnt}_{hj}-\sum_{h=1}^{N} \theta_{h}\mathrm{lnt}_{jh}+\sum_{j=1}^{N} \sum_{h=1}^{N} \theta_{h}\theta_{j}\mathrm{lnt}_{hj}.........(5)$$

$$\theta_{j}=\frac{y_{h}}{y_{w}}..............(6)$$

Baier & Bergstrand (2009) recommended to derive trade cost term for each trade cost variable in the model. Therefore our trade cost variable, adjusted for MR, can be expressed as

$$\ln Dist_{ij}*=\ln Dist_{hj}-\sum_{j=1}^{N} \theta_{j}\ln Dist_{hj}-\sum_{h=1}^{N} \theta_{h}\ln Dist_{jh}+\sum_{j=1}^{N} \sum_{h=1}^{N} \theta_{h}\theta_{j}\ln Dist_{hj}.........(7)$$

$$\ln\mathrm{Im}t_{hj}*=\ln\mathrm{Im}t_{hj}-\sum_{j=1}^{N} \theta_{j}\ln\mathrm{Im}t_{hj}-\sum_{h=1}^{N} \theta_{h}\ln\mathrm{Im}t_{jh}+\sum_{j=1}^{N} \sum_{h=1}^{N} \theta_{h}\theta_{j}\ln\mathrm{Im}t_{hj}.........(8)$$

$$Con_{hj}*=Con_{hj}-\sum_{j=1}^{N} \theta_{j}Con_{hj}-\sum_{h=1}^{N} \theta_{h}Con_{jh}+\sum_{j=1}^{N} \sum_{h=1}^{N} \theta_{h}\theta_{j}Con_{hj}.........(9)$$

$$Lang_{hj}*=Lang_{hj}-\sum_{j=1}^{N} \theta_{j}Lang_{hj}-\sum_{h=1}^{N} \theta_{h}Lang_{jh}+\sum_{j=1}^{N} \sum_{h=1}^{N} \theta_{h}\theta_{j}Lang_{hj}.........(10)$$

$$Pta_{hj}*=Pta_{hj}-\sum_{j=1}^{N} \theta_{j}Pta_{hj}-\sum_{h=1}^{N} \theta_{h}Pta_{jh}+\sum_{j=1}^{N} \sum_{h=1}^{N} \theta_{h}\theta_{j}Pta_{hj}.........(11)$$

$$Col_{hj}*=Col_{hj}-\sum_{j=1}^{N} \theta_{j}Col_{hj}-\sum_{h=1}^{N} \theta_{h}Col_{jh}+\sum_{j=1}^{N} \sum_{h=1}^{N} \theta_{h}\theta_{j}Col_{hj}.........(12)$$

$$Pd_{hj}*=Pd_{hj}-\sum_{j=1}^{N} \theta_{j}Pd_{hj}-\sum_{h=1}^{N} \theta_{h}Pd_{jh}+\sum_{j=1}^{N} \sum_{h=1}^{N} \theta_{h}\theta_{j}Pd_{hj}.........(13)$$
